# ﻿Phylogeny of *Phyllozyma* (Spiculogloeaceae, Spiculogloeales) with description of four new species from China

**DOI:** 10.3897/mycokeys.123.161540

**Published:** 2025-10-15

**Authors:** Zhi-Wen Xi, Chun-Yue Chai, Qiu-Hong Niu, Feng-Li Hui

**Affiliations:** 1 School of Life Science, Nanyang Normal University, Nanyang 473061, China Nanyang Normal University Nanyang China; 2 Research Center of Henan Provincial Agricultural Biomass Resource Engineering and Technology, Nanyang Normal University, Nanyang 473061, China Nanyang Normal University Nanyang China

**Keywords:** Basidiomycetes, phylogenetic analysis, phylloplane yeast, taxonomy

## Abstract

*Phyllozyma*, belonging to the family Spiculogloeaceae of the order Spiculogloeales, is a genus of blastoconidia-forming yeasts. Until now, nine *Phyllozyma* species have been described. During our investigation of yeast diversity in China, several *Phyllozyma* strains were isolated from the surface of plant leaves collected in Guizhou and Hainan provinces, which represent undescribed taxa. Based on multi-locus (ITS, LSU, *TEF1*, and *RBP1*) and single-locus (ITS) phylogenetic analyses, as well as phenotypic characteristics, these strains were identified as four new species of *Phyllozyma*: *P. aucubae***sp. nov.** (holotype CICC 33627^T^), *P. camelliae***sp. nov.** (holotype CICC 33625^T^), *P. diaoluoensis***sp. nov.** (holotype CICC 33620^T^), and *P. guizhouensis***sp. nov.** (holotype CICC 33628^T^). *P. aucubae***sp. nov.** was identified as a nonballistoconidium-forming species. This phenomenon is extremely rare in the genus *Phyllozyma*, and prior to this report, only *P. jiayinensis* was reported to lack the ability to produce ballistoconidia.

## ﻿Introduction

Spiculogloeales was established by Bauer et al. (2006), originally comprising two teleomorphic genera, *Spiculogloea* and *Mycogloea*, along with one anamorphic genus, *Sporobolomyces*. Among them, *Sporobolomyces* represented the largest genus, encompassing over 50 species as of earlier reports (Hamamoto et al. 2011). However, molecular phylogenetic analyses based on the D1/D2 domain of the large subunit (LSU) rRNA gene (Fell et al. 2000; Scorzetti et al. 2002; Furuya et al. 2012), the small subunit (SSU) rRNA gene (Hamamoto and Nakase 2000), and the internal transcribed spacer (ITS) region (Scorzetti et al. 2002) demonstrated that *Sporobolomyces* is a polyphyletic taxon. To address this polyphyly, Wang et al. (2015) reclassified seven *Sporobolomyces* species from the subbrunneus clade into a newly proposed genus, *Phyllozyma*. These species—*P. coprosmicola*, *P. corallina*, *P. dimennae*, *P. linderae*, *P. novozealandica*, *P. producta*, and *P. subbrunnea*—were segregated based on multigene phylogenetic analyses involving seven loci: SSU, ITS, LSU, *RPB1*, *RPB2*, *TEF1*, and *CYTB*, along with a revised LSU dataset. Among these, *P. subbrunnea* was designated as the type species of the newly circumscribed genus (Wang et al. 2015). More recently, two additional species, *P. aceris* and *P. jiayinensis*, were described from phylloplane habitats—specifically, from *Acer caudatum* and an unidentified plant species collected in China, respectively (Li et al. 2020).

All currently known species of the genus *Phyllozyma* are represented solely by their asexual yeast forms, characterized morphologically by polar budding as the mode of propagation. Most species may form ballistoconidia, and some species may also form hyphae and pseudohyphae (Hamamoto et al. 2011; Wang et al. 2015). Physiologically, all members of the genus lack fermentative ability, possess Q-10 as a predominant ubiquinone, and assimilate various carbon sources, but not maltose, melezitose, L-arabinose, or myo-inositol (Hamamoto et al. 2011; Wang et al. 2015; Li et al. 2020). *Phyllozyma* species are associated with plant leaves (Nakase and Suzuki 1985; Hamamoto and Nakase 1995; Nakase et al. 1994; Furuya et al. 2012; Li et al. 2020) and are ecologically distinct from the teleomorphic species in the genera *Spiculogloea* and *Mycogloea*, which function as mycoparasites and are characterized by tremelloid haustorial cells (Roberts 1996; Bauer 2004; Weiß et al. 2004; Wang et al. 2015).

Until now, nine *Phyllozyma* species have been accepted, and they are mainly distributed in temperate and subtropical regions, especially in Asia (Nakase and Suzuki 1985; Nakase et al. 1994; Furuya et al. 2012; Li et al. 2020). In China, *P. corallina* and *P. producta* were first reported in Zhejiang Province in 2018 (Zang et al. 1998). Later, six additional species, including the newly identified *P. aceris* and *P. jiayinensis*, were discovered in Jilin Province and the Tibet Autonomous Region (Li et al. 2020). China’s vast temperate regions in the Northern Hemisphere likely host a diverse array of *Phyllozyma* species, yet they are poorly documented. In this study, we isolated nine *Phyllozyma* strains from Guizhou and Hainan provinces, China. Molecular phylogenetic analyses combined with phenotypic characterization revealed that they represent four previously undescribed species. The aim of this investigation is to apply an integrative taxonomic approach for the identification and description of these new taxa.

## ﻿Materials and methods

### ﻿Sample collection and yeast isolation

Leaf samples were collected in Guizhou and Hainan provinces, China. Yeast strains were isolated from the leaf surfaces using the improved ballistospore-fall method described by Nakase and Takashima (1993). Briefly, fresh leaves were cut into small pieces and adhered with a thin layer of petroleum jelly to the inner lid of a Petri dish containing yeast malt (YM) agar. YM agar consisted of 0.3% yeast extract, 0.3% malt extract, 0.5% peptone, 1% glucose, and 2% agar and was supplemented with 0.01% chloramphenicol to prevent bacterial growth. Plates were incubated at 20 °C and monitored daily for colony formation. Selected colonies were streaked back onto YM agar plates for purification. Following purification, strains were suspended in 20% (v/v) glycerol and stored at −80 °C for long-term preservation.

### ﻿Phenotypic characterization

Morphological, physiological, and biochemical characterizations were conducted according to standardized methods established by Kurtzman et al. (2011). Glucose fermentation was tested in liquid medium using Durham fermentation tubes. Carbon and nitrogen assimilation capabilities were examined in liquid medium, with starved inoculum used for nitrogen assimilation testing (Kurtzman et al. 2011). Cell morphology was examined with a Leica DM2500 microscope (Leica, Wetzlar, Germany) and LAS v.4.13 software. Ballistoconidium-forming activity was investigated using the inverted-plate method (do Carmo-Sousa and Phaff 1962) after 2 weeks of incubation on cornmeal agar (CMA; 2.5% cornstarch and 2% agar) at 20 °C. Growth at various temperatures (15, 20, 25, 30, 35, and 37 °C) was assessed through cultivation on YM agar plates. The potential sexual cycle of each strain was investigated on CMA, potato dextrose agar (PDA; 20% potato infusion, 2% glucose, and 2% agar), and V8 agar (10% V8 juice and 2% agar). Each tested strain was inoculated on agar plates and incubated at 20 °C for up to 2 months, with observations made every 2 weeks (Li et al. 2020). All novel taxonomic descriptions and proposed names were deposited in the MycoBank database (http://www.mycobank.org; accessed 8 January 2025).

### ﻿DNA extraction, PCR amplification, and sequencing

Genomic DNA was extracted from each strain using the Ezup Column Yeast Genomic DNA Purification Kit according to the manufacturer’s instructions (Sangon Biotech Co., Shanghai, China). The internal transcribed spacer (ITS) region, the D1/D2 domain of the large subunit (LSU) rRNA gene, the translation elongation factor 1-α gene (*TEF1*), and the RNA polymerase II largest subunit (*RPB1*) were amplified with primers ITS1/ITS4 (White et al. 1990), NL1/NL4 (Kurtzman and Robnett 1998), EF1-526F/EF1-1567R (Rehner and Buckley 2005), and RPB1-Af/RPB1-Cr (Matheny et al. 2002), respectively. The PCR products were checked in a 1% (w/v) agarose gel, purified using a SanPrep Column PCR Product Purification Kit (Sangon Biotech, Shanghai, China), and sequenced using an ABI 3730xl DNA analyzer with the same primers used for PCR amplification. The identity and accuracy of each sequence were verified by comparison with sequences in the GenBank database. Assembly was performed with BioEdit v.7.1.3.0 (Hall 1999). All newly generated sequences were deposited in the GenBank database (https://www.ncbi.nlm.nih.gov/genbank/).

### ﻿Phylogenetic analysis

The sequences generated in this study, along with additional sequences downloaded from the GenBank database (Table 1), were used in phylogenetic analyses. Species of *Spiculogloea* were not included in the phylogenetic analysis, except for *Spiculogloea* sp. DB 1496, because sequence data for the type species of this genus are presently not available in public databases. Following Li et al. (2020), *Mixia osmundae* CBS 9802 was selected as the outgroup. The combined dataset of ITS, LSU, *TEF1*, and *RPB1* was used to explore the phylogenetic positions of the newly isolated strains within *Phyllozyma*. The ITS dataset was then used to further differentiate species identities within this genus. Sequences from each locus were first aligned individually using MAFFT v.7.110 (Katoh and Standley 2013) with the G-INS-i option. Poorly aligned regions were manually removed using MEGA v.11 (Tamura et al. 2021). The aligned sequences from different loci were concatenated with PhyloSuite v.1.2.2 (Zhang et al. 2020).

**Table 1.** List of species, strains, and GenBank accession numbers of sequences used in this study.

Species	Strain number	Locality	GenBank accession no.				References
			ITS	LSU D1/D2	RPB1	TEF1	
*Meniscomyces layueensis*	CGMCC 2.5681^T^	China	MK050380	MK050380	MK849248	MK849112	Li et al. 2020
*Meniscomyces senecionis*	PYCC 9960^T^	China	OR035763	OP954745	–	–	Li et al. 2020
*Phyllozyma aceris*	CGMCC 2.2662^T^	China	NR_175625	MK050377	MK849136	MK849006	Li et al. 2020
*Phyllozyma aceris*	CGMCC 2.2617^T^	China	MK050378	MK050378	MK849132	–	Li et al. 2020
***Phyllozyma aucubae***	**NYNU 239180^T^**	**China**	**OR961460**	**OR958754**	**PX353021**	**PV654547**	**This study**
***Phyllozyma aucubae***	**NYNU 239198**	**China**	**PP660918**	**PP660917**	–	**PV654548**	**This study**
***Phyllozyma camelliae***	**NYNU 23731^T^**	**China**	**PP033661**	**PP033657**	**PX353019**	**PV654542**	**This study**
***Phyllozyma camelliae***	**NYNU 24899**	**China**	**PQ899973**	**PQ899972**	**PX353020**	**PV654543**	**This study**
*Phyllozyma coprosmicola*	CBS 7897^T^	New Zealand	NR_073316	NG_058371	–	KJ707908	Hamamoto et al. 1995
*Phyllozyma corallina*	MAFF 654003^T^	Japan	AB638335	AB638335	–	–	Furuya et al. 2012
***Phyllozyma diaoluoensis***	**NYNU 2377^T^**	**China**	**OR526726**	**OR511464**	**PX353016**	**PV654544**	**This study**
***Phyllozyma diaoluoensis***	**NYNU 23732**	**China**	**OR961462**	**OR958779**	**PX353017**	**PV654545**	**This study**
***Phyllozyma diaoluoensis***	**NYNU 23718**	**China**	**OR958777**	**OR958778**	**PX353018**	**PV654546**	**This study**
*Phyllozyma dimennae*	JCM 8762^T^	New Zealand	NR_144764	AB644404	KJ707991	KJ707907	Hamamotoet al. 1995
***Phyllozyma guizhouensis***	**NYNU 239199^T^**	**China**	**OR958770**	**OR958769**	–	**PV654549**	**This study**
***Phyllozyma guizhouensis***	**NYNU 248104**	**China**	**PQ899975**	**PQ899974**	–	**PV654550**	**This study**
*Phyllozyma jiayinensis*	CGMCC 2.5669^T^	China	MK050376	MK849108	–	MK849108	Li et al. 2020
*Phyllozyma linderae*	CBS 7893^T^	Japan	NR_073319	AF189989	–	KJ707906	Nakase et al. 1994
*Phyllozyma novozealandica*	JCM 8756 ^T^	New Zealand	NR_144765	KJ708467	KJ708073	KJ707851	Hamamotoet al. 1995
*Phyllozyma producta*	MAFF 654001^T^	Japan	AB638334	AB638334	–	–	Furuya et al. 2012
*Phyllozyma subbrunnea*	CBS 7196^T^	Japan	NR_077094	AF189997	–	KJ707909	Nakase and Suzuki 1985
*Sporobolomyces* sp.	TY-285	Japan	AY313080	AY313059	–	–	–
*Spiculogloea* sp.	DB 1496	Germany	–	AY512885	–	–	–
Uncultured basidiomycete yeast	TFL3-16	China	AJ582959	–	–	–	–
*Mixia osmundae*	CBS 9802^T^	Sezawa	DQ831010	DQ831009	KJ708076	KJ707837	Sjamsuridzal et al. 2002

^T^ , type strain. Species obtained in this study are in bold.

Maximum likelihood (ML) and Bayesian inference (BI) analyses were performed with RAxML v.8.2.3 (Stamatakis 2014) and MrBayes v.3.1.2 (Ronquist and Huelsenbeck 2003), respectively. The best nucleotide substitution model was estimated using Modeltest v.3.04 (Posada and Crandall 1998). In the ML analyses, bootstrap (BS) values were assessed through 1,000 rapid bootstrap replicates. For BI analyses, six Markov chain Monte Carlo (MCMC) chains were run simultaneously for 50 million generations, and trees were sampled every 1,000 generations. The first 25% of the generated trees were discarded as burn-in, and the remaining trees were used to estimate Bayesian posterior probabilities (BPPs) for the clades.

## ﻿Results

### ﻿Molecular phylogeny

Among the yeasts isolated from leaf samples collected across different regions of China, nine strains identified as *Phyllozyma* based on their rRNA gene sequences were selected for further phylogenetic studies.

**Figure 1.**
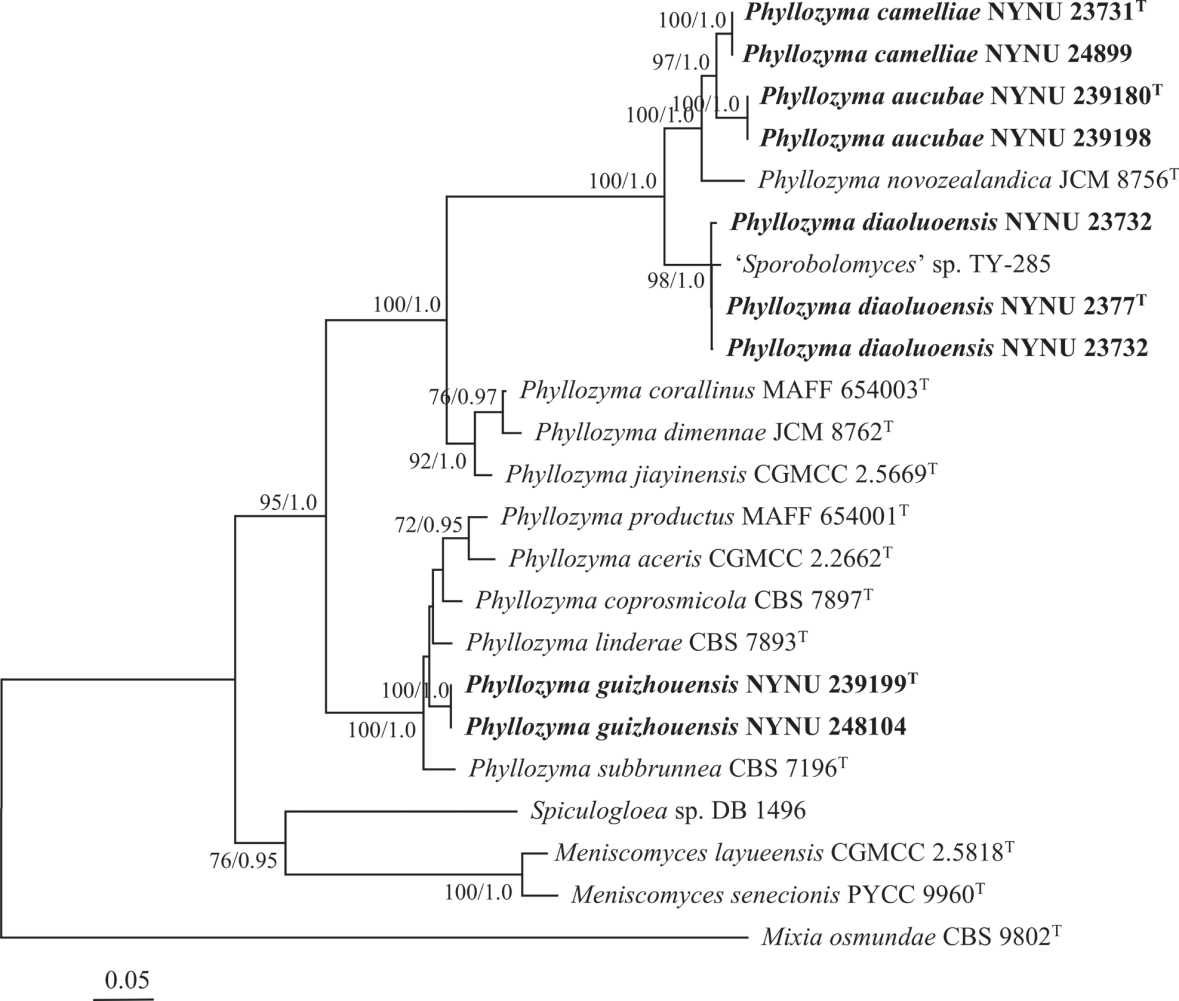
Phylogenetic positions of the newly studied strains of *Phyllozyma* inferred from the combined dataset of ITS, LSU, *TEF1*, and *RPB1*. The topology generated by the maximum likelihood method is presented along with bootstrap values and Bayesian posterior probabilities above 50% and 0.95, respectively, at the nodes. The tree is rooted with *Mixia osmundae* CBS 9802. Type strain sequences are marked with superscript ^T^. New species are highlighted in bold.

The combined dataset of ITS, LSU, *TEF1*, and *RPB1* from 60 sequences generated a concatenated alignment of 2,368 characters (543 characters from ITS, 638 characters from LSU, 504 characters from *TEF1*, and 693 characters from *RPB1*) with GTR+I+G as the best-fit evolutionary model. ML and BI methods generated similar topologies in the main lineages; therefore, only the topology generated by the ML method is presented, along with BS values and BPPs above 50% and 0.95, respectively, at the nodes (Fig. 1). The phylogeny generated from this dataset strongly supported *Phyllozyma* as a monophyletic genus (BS = 95%, BPP = 1.0). The nine newly isolated strains formed four well-supported groups distinct from other species of *Phyllozyma*.

**Figure 2.**
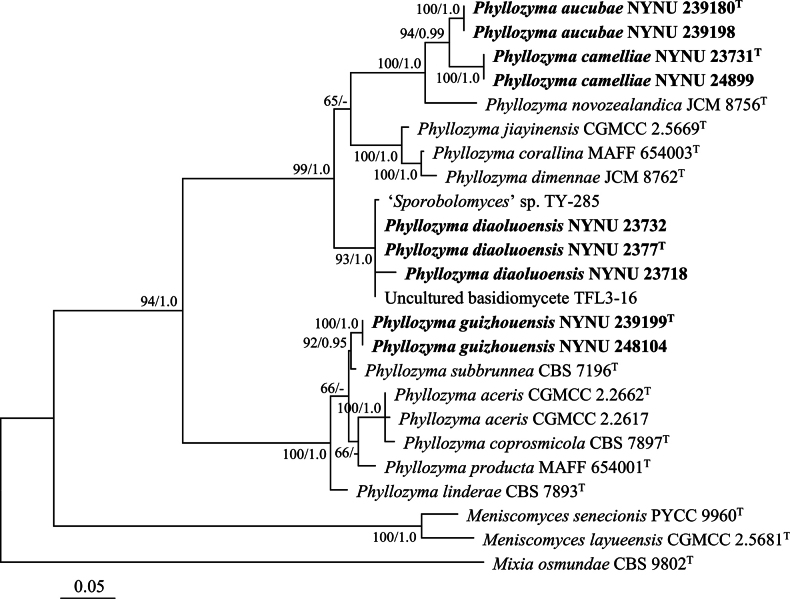
Species identities of *Phyllozyma* differentiated by ITS-based phylogeny. The tree generated by the maximum likelihood method is presented along with bootstrap values and Bayesian posterior probabilities above 50% and 0.95, respectively, at the nodes. The tree is rooted with *Mixia osmundae* CBS 9802. Type strain sequences are marked with superscript ^T^. New species are highlighted in bold.

The ITS dataset of *Phyllozyma*, comprising 24 sequences, generated an alignment of 543 characters with GTR+I+G as the best-fit evolutionary model. ML and BI methods produced similar topologies in the main lineages; therefore, the topology inferred from the ML method is presented, along with BS values and BPPs above 50% and 0.95, respectively, at the nodes (Fig. 2). This tree recovered nine known species of *Phyllozyma*, while the newly studied strains formed four independent groups, consistent with the phylogeny inferred from the combined ITS, LSU, *TEF1*, and *RPB1* dataset.

Groups NYNU 23731 and NYNU 239180, each containing two strains, clustered in the same clade as *P. novozealandica* in the trees constructed with all datasets (Figs 1, 2). Strains in the NYNU 23731 group had identical ITS and D1/D2 sequences, indicating that they are conspecific. Similarly, strains in the NYNU 239180 group shared identical ITS and D1/D2 sequences, which differed from those of the NYNU 23731 group by four nucleotides (nt) (~0.7%) in the D1/D2 domain and 12 nt (~2.4%) in the ITS region. In addition, these two groups differed from their closest known species, *P. novozealandica*, by 10–14 nt (~1.7–2.4%) in the D1/D2 domain and 25–29 nt (~5.0–5.9%) in the ITS region. These results suggest that groups NYNU 23731 and NYNU 239180 represent two novel species of *Phyllozyma*.

The group NYNU 2377, consisting of three strains, clustered together with an unpublished strain, ‘*Sporobolomyces*’ sp. TY-285 (Fig. 1), and clone TFL3-16, an unculturable basidiomycete (Fig. 2). These five strains possessed similar ITS and D1/D2 sequences, with no more than four nucleotide differences, suggesting that they are conspecific. The NYNU 2377 group differed from its closest related species, *P. jiayinensis*, by 31–32 nt (~5.2–5.5%) in the D1/D2 domain and 52–65 nt (~8.9–9.4%) in the ITS region. These results strongly suggest that the NYNU 2377 group represents another novel species of *Phyllozyma*.

The group NYNU 239199, containing two strains, had identical ITS and D1/D2 sequences, indicating that they are conspecific. Strains in the NYNU 239199 group formed a separate subclade from other *Phyllozyma* species in the tree of the multilocus dataset (Fig. 1) but clustered with *P. subbrunnea* with high support in the tree of the ITS dataset (Fig. 2). This group differed from its closest related species, *P. subbrunnea*, by 13 nt (~2.2%) in the D1/D2 domain and 7 nt (~1.4%) in the ITS region. These results indicate that the NYNU 239199 group belongs to a novel species of *Phyllozyma*.

### ﻿Taxonomy

#### ﻿ Phyllozyma aucubae

Taxon classificationFungiSpiculogloealesSpiculogloeaceae

Z.W. Xi & F.L. Hui sp. nov.

B32E7C0B-0FA2-5104-B054-4296EEEA8838

MB 857393

Fig. 3

##### Etymology.

The specific epithet “*aucubae*” refers to *Aucuba*, the plant genus from which the type strain was isolated.

##### Typus.

China • Guizhou Prov.: Guiyang City, East Mountain Park, in the phylloplane of *Aucuba japonica*, 15 Sept 2023, D. Lu, NYNU 239180 (holotype CICC 33627^T^ preserved as a metabolically inactive state, culture ex-type PYCC 9993).

##### Description.

On YM agar after 7 days at 20 °C, the streak culture is cream, mucoid, smooth, and glistening, with an entire margin. After 3 days in YM broth at 20 °C, cells are cylindrical, 2.1–2.2 × 6.4–11.9 μm and single, budding is polar. After 1 month at 20 °C, a ring and sediment are present. In Dalmau plate culture on CMA, simple pseudohyphae and formed. Sexual structures are not observed on PDA, CMA, or V8 agar. Ballistoconidia are not produced. Glucose fermentation is absent. Glucose, inulin (weak), D-arabinose (delayed), glycerol (delayed), D-mannitol, D-glucitol (delayed), DL-lactate (delayed and weak), and succinate (delayed) are assimilated as sole carbon sources. Sucrose, raffinose, melibiose, galactose, lactose, trehalose, maltose, melezitose, methyl-α-D-glucoside, cellobiose, salicin, L-sorbose, L-rhamnose, D-xylose, L-arabinose, 5-keto-D-gluconate, D-ribose, methanol, ethanol, erythritol, ribitol, galactitol, myo-inositol, citrate, D-gluconate, D-glucosamine, N-acetyl-D-glucosamine, 2-keto-D-gluconate, D-glucuronate, and glucono-1,5-lactone are not assimilated. Nitrate (delayed and weak), nitrite (delayed and weak), ethylamine (delayed), and L-lysine are assimilated as sole nitrogen sources. Cadaverine is not assimilated. Maximum growth temperature is 25 °C. Growth on 50% (w/w) glucose-yeast extract agar is negative. Starch-like substances are not produced. Urease activity is positive. Diazonium Blue B reaction is positive.

**Figure 3.**
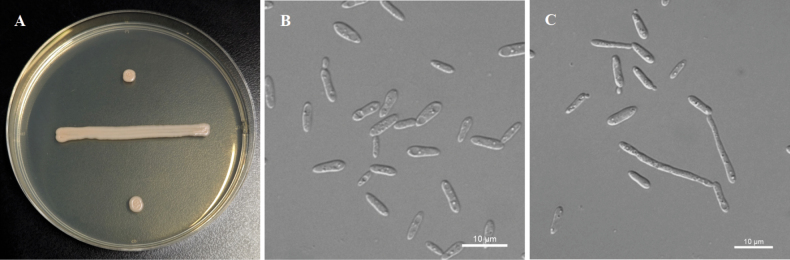
Morphological characteristics of *P. aucubae* sp. nov. NYNU 239180^T^. A. The streak culture grown on YM agar after 7 d at 20 °C; B. Budding cells grown in YM broth for 3 d at 20 °C; C. Simple pseudohyphae produced on CMA after 7 d at 20 °C. Scale bars: 10 μm.

##### Additional strain examined.

China • Guizhou Prov.: Guiyang City, East Mountain Park, in the phylloplane of *Aucuba japonica*, 15 Sept 2023, D. Lu, NYNU 239198.

##### GenBank accession numbers.

Holotype CICC 33627^T^ (ITS: OR961460, D1/D2: OR958754, *RPB1*: PX353021, *TEF1*: PV654547); additional strain NYNU 239198 (ITS: PP660918, D1/D2: PP660917, *TEF1*: PV654548).

##### Note.

Physiologically, *P. aucubae* sp. nov. differs from its closely related species, *P. camelliae* sp. nov., described in this study, by its inability to assimilate galactose and trehalose, as well as its ability to grow at 30 °C (Table 2).

**Table 2.** Physiological and biochemical characteristics that differentiate the new species and their closest related species.

Characteristics	1	2	3	4*	5	6*
Carbon assimilation						
Inulin	w	d/w	+	–	+	–
Sucrose	–	–	+	–	+	+
Raffinose	–	–	d	–	d	+
Galactose	–	d	+	–	–	–
Trehalose	–	+	d	+	+	+
D-Arabinose	d	d	w	–	d	+
Glycerol	d	d	+	–	+	+
Ribitol	–	–	w	–	d/w	+
DL-Lactate	d/w	d/w	–	w	+	+
Succinate	d	d/w	w	–	+	+
Citrate	–	–	w	–	–	+
Nitrogen assimilation						
Nitrite	d/w	d	d	–	d/w	+
Ethylamine	d	d	–	–	–	–
L-Lysine	+	+	–	–	+	w
Growth tests						
Growth at 30 °C	–	+	+	–	+	–

Species: 1, *P. aucubae* sp. nov.; 2, *P. camelliae* sp. nov.; 3, *P. diaoluoensis* sp. nov.; 4, *P. jiayinensis*; 5, *P. guizhouensis* sp. nov.; 6, *P. subbrunnea*. +, positive reaction; –, negative reaction; d, delayed positive; w, weakly positive. All data were produced in this study, except where indicated with an asterisk (*), which were obtained from the original description (Hamamoto et al. 2011; Li et al. 2020).

#### ﻿ Phyllozyma camelliae

Taxon classificationFungiSpiculogloealesSpiculogloeaceae

Z.W. Xi & F.L. Hui sp. nov.

C44EC236-F12A-5CC5-986E-2C18D8521355

MB 857394

Fig. 4

##### Etymology.

The specific epithet “*camelliae*” refers to *Camellia*, the plant genus from which the type strain was isolated.

##### Typus.

China • Hainan Prov.: Qiongzhong Li and Miao Autonomous County, Diaoluo Mountain, in the phylloplane of *Camellia oleifera*, 15 Jul 2023, X.M. Han, NYNU 23731 (holotype CICC 33625^T^ preserved as a metabolically inactive state, culture ex-type PYCC 9991).

##### Description.

On YM agar after 7 days at 20 °C, the streak culture is pale-yellow, mucoid, smooth, and glistening, with an entire margin. After 3 days in YM broth at 20 °C, cells are cylindrical, 1.5–2.4 × 5.3–8.5 μm and single, budding is polar. After 1 month at 20 °C, a ring and sediment are present. In Dalmau plate culture on CMA, pseudohyphae and hyphae are not formed. Sexual structures are not observed on PDA, CMA, or V8 agar. Ballistoconidia are ellipsoidal, 2.0–2.7 × 2.7–4.3 μm. Glucose fermentation is absent. Glucose, inulin (delayed and weak), galactose (delayed), trehalose, D-arabinose (delayed), glycerol (delayed), D-mannitol, D-glucitol (delayed), DL-lactate (delayed and weak), and succinate (delayed and weak) are assimilated as sole carbon sources. Sucrose, raffinose, melibiose, lactose, maltose, melezitose, methyl-α-D-glucoside, cellobiose, salicin, L-sorbose, L-rhamnose, D-xylose, L-arabinose, 5-keto-D-gluconate, D-ribose, methanol, ethanol, erythritol, ribitol, galactitol, myo-inositol, citrate, D-gluconate, D-glucosamine, N-acetyl-D-glucosamine, 2-keto-D-gluconate, D-glucuronate, and glucono-1,5-lactone are not assimilated. Nitrate, nitrite (delayed), ethylamine (delayed), and L-lysine are assimilated as sole nitrogen sources. Cadaverine is not assimilated. Maximum growth temperature is 30 °C. Growth on 50% (w/w) glucose-yeast extract agar is negative. Starch-like substances are not produced. Urease activity is positive. Diazonium Blue B reaction is positive.

**Figure 4.**
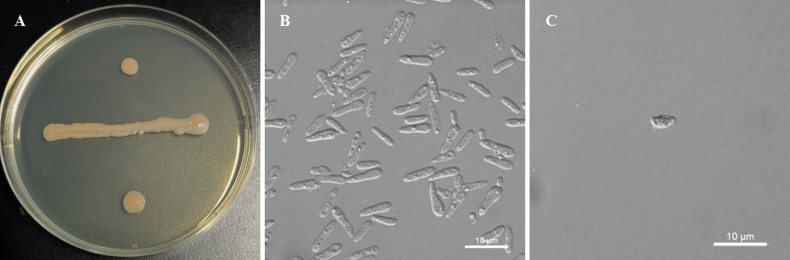
Morphological characteristics of *P. camelliae* sp. nov. NYNU 23731^T^. A. The streak culture grown on YM agar after 7 d at 20 °C; B. Budding cells grown in YM broth for 3 d at 20 °C; C. Ballistoconidia produced on CMA after 7 d at 20 °C. Scale bars: 10 μm.

##### Additional strain examined.

China • Hainan Prov.: Wuzhishan City, Wuzhi Mountain, in the phylloplane of *Symplocos adenophylla*, 12 Aug 2024, Y.F. Lu & F.L. Hui, NYNU 24899.

##### GenBank accession numbers.

Holotype CICC 33625^T^ (ITS: PP033661, D1/D2: PP033657, *RPB1*: PX353019, *TEF1*: PV654542); additional strain NYNU 24899 (ITS: PP239073, D1/D2: PP239062, *RPB1*: PX353020, *TEF1*: PV654543).

##### Note.

See the “Notes” of the previous species.

#### ﻿ Phyllozyma diaoluoensis

Taxon classificationFungiSpiculogloealesSpiculogloeaceae

Z.W. Xi & F.L. Hui sp. nov.

50A1A233-BEAE-552B-85D4-B87033547B4F

MB 857395

Fig. 5

##### Etymology.

The specific epithet “*diaoluoensis*” refers to the geographic origin of the type strain, Diaoluo Mountain, Qiongzhong Li and Miao Autonomous County, Hainan Province.

##### Typus.

China • Hainan Prov.: Qiongzhong Li and Miao Autonomous County, Diaoluo Mountain, in the phylloplane of *Zanthoxylum avicennae*, 15 Jul 2023, X.M. Han, NYNU 2377 (holotype CICC 33620^T^ preserved as a metabolically inactive state, culture ex-type PYCC 9983).

##### Description.

On YM agar after 7 days at 20 °C, the streak culture is pale-yellow, tough, and somewhat wrinkled, with an entire margin. After 3 days in YM broth at 20 °C, cells are cylindrical, 2.0–2.8 × 4.3–6.8 μm and single, budding is polar. After 1 month at 20 °C, a ring and sediment are present. In Dalmau plate culture on CMA, pseudohyphae and hyphae are not formed. Sexual structures are not observed on PDA, CMA, or V8 agar. Ballistoconidia are falcate or cylindrical, 2.5–4.9 × 8.7–12.6 μm. Glucose fermentation is absent. Glucose, inulin, sucrose, raffinose (delayed), galactose, trehalose (delayed), D-arabinose (weak), glycerol, ribitol (weak), D-mannitol (weak), D-glucitol, succinate (weak), citrate (weak), and D-glucuronate (weak) are assimilated as sole carbon sources. Melibiose, lactose, maltose, melezitose, methyl-α-D-glucoside, cellobiose, salicin, L-sorbose, L-rhamnose, D-xylose, L-arabinose, 5-keto-D-gluconate, D-ribose, methanol, ethanol, erythritol, galactitol, myo-inositol, DL-lactate, D-gluconate, D-glucosamine, N-acetyl-D-glucosamine, 2-keto-D-gluconate, and D-glucono-1,5-lactone are not assimilated. Nitrate (delayed) and nitrite (delayed) are assimilated as sole nitrogen sources. Ethylamine, L-lysine, and cadaverine are not assimilated. Maximum growth temperature is 30 °C. Growth on 50% (w/w) glucose-yeast extract agar is negative. Starch-like substances are not produced. Urease activity is positive. Diazonium Blue B reaction is positive.

**Figure 5.**
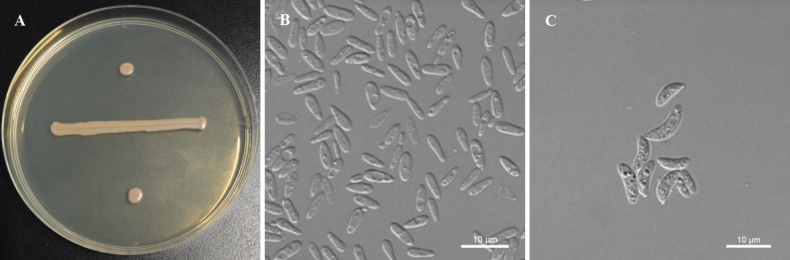
Morphological characteristics of *P. diaoluoensis* sp. nov. NYNU 2377^T^. A. The streak culture grown on YM agar after 7 d at 20 °C; B. Budding cells grown in YM broth for 3 d at 20 °C; C. Ballistoconidia produced on CMA after 7 d at 20 °C. Scale bars: 10 μm.

##### Additional strain examined.

China • Hainan Prov.: Qiongzhong Li and Miao Autonomous County, Diaoluo Mountain, in the phylloplane of *Gordonia hainanensis*, 15 Jul 2023, X.M. Han, NYNU 23732; in the phylloplane of *Altingia obovata*, 15 Jul 2023, X.M. Han, NYNU 23718.

##### GenBank accession numbers.

Holotype CICC 33620^T^ (ITS: OR526726, D1/D2: OR511464, *RPB1*: PX353016, *TEF1*: PV654544); additional strains NYNU 23732 (ITS: OR961462, D1/D2: OR958779, *RPB1*: PX353017, *TEF1*: PV654545) and NYNU 23718 (ITS: OR958777, D1/D2: OR958778, *RPB1*: PX353018, *TEF1*: PV654546).

##### Note.

Physiologically, *P. diaoluoensis* sp. nov. differs from its closely related species *P. jiayinensis* in its ability to assimilate inulin, sucrose, raffinose, galactose, trehalose, D-arabinose, glycerol, ribitol, succinate, and citrate and its inability to assimilate DL-Lactate. Furthermore, *P. diaoluoensis* sp. nov. can grow at 30 °C, while *P. jiayinensis* cannot (Table 2).

#### ﻿ Phyllozyma guizhouensis

Taxon classificationFungiSpiculogloealesSpiculogloeaceae

Z.W. Xi & F.L. Hui sp. nov.

45031B87-3B08-5632-9A3E-9FF6EEF343E4

MB 857396

Fig. 6

##### Etymology.

The specific epithet “*guizhouensis*” refers to the geographic origin of the type strain, East Mountain Park, Guiyang City, Guizhou Province.

##### Typus.

China • Guizhou Prov.: Guiyang City, East Mountain Park, in the phylloplane of *Aucuba japonica*, 15 Sept 2023, D. Lu, NYNU 239199 (holotype CICC 33628^T^ preserved as a metabolically inactive state, culture ex-type PYCC 9994).

##### Description.

On YM agar after 7 days at 20 °C, the streak culture is pale-yellow, butyrous, smooth, and glossy, with an entire margin. After 3 days in YM broth at 20 °C, cells are cylindrical, 2.2–3.5 × 7.0–10.4 μm and single, budding is polar. After 1 month at 20 °C, a ring and sediment are present. In Dalmau plate culture on CMA, pseudohyphae and hyphae are not formed. Sexual structures are not observed on PDA, CMA, or V8 agar. Ballistoconidia are falcate or cylindrical, 2.8–3.4 × 7.9–9.6 μm. Glucose fermentation is absent. Glucose, inulin, sucrose, raffinose (delayed), trehalose, D-arabinose (delayed), glycerol, ribitol (delayed and weak), D-mannitol, D-glucitol (delayed), DL-lactate, and succinate are assimilated as sole carbon sources. Melibiose, galactose, lactose, maltose, melezitose, methyl-α-D-glucoside, cellobiose, salicin, L-sorbose, L-rhamnose, D-xylose, L-arabinose, 5-keto-D-gluconate, D-ribose, methanol, ethanol, erythritol, galactitol, myo-inositol, citrate, D-gluconate, D-glucosamine, N-acetyl-D-glucosamine, 2-keto-D-gluconate, D-glucuronate, and D-glucono-1,5-lactone are not assimilated. Nitrate, nitrite (delayed and weak), and L-lysine are assimilated as sole nitrogen sources. Ethylamine and cadaverine are not assimilated. Maximum growth temperature is 30 °C. Growth on 50% (w/w) glucose-yeast extract agar is negative. Starch-like substances are not produced. Urease activity is positive. Diazonium Blue B reaction is positive.

**Figure 6.**
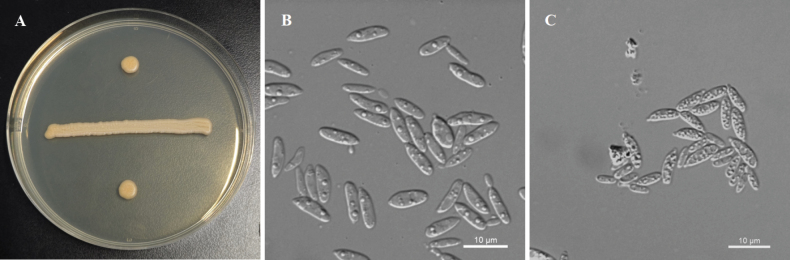
Morphological characteristics of *P. guizhouensis* sp. nov. NYNU 239199^T^. A. The streak culture grown on YM agar after 7 d at 20 °C; B. Budding cells grown in YM broth for 3 d at 20 °C; C. Ballistoconidia produced on corn meal agar after 7 d at 20 °C. Scale bars: 10 μm.

##### Additional strain examined.

China • Hainan Prov.: Wuzhishan City, Wuzhi Mountain, in the phylloplane of *Ervatamia divaricata*, 12 Aug 2024, Y.F. Lu & F.L. Hui, NYNU 248104.

##### GenBank accession numbers.

Holotype CICC 33628^T^ (ITS: OR958770, D1/D2: OR958769, *TEF1*: PV654549); additional strain NYNU 248104 (ITS: OR961462, D1/D2: OR958779, *TEF1*: PV654550).

##### Note.

Physiologically, *P. guizhouensis* sp. nov. differs from its closely related species *P. subbrunnea* in its ability to assimilate inulin and its inability to assimilate citrate. Furthermore, *P. guizhouensis* sp. nov. can grow at 30 °C, while *P. subbrunnea* cannot (Table 2).

## ﻿Discussion

In the present study, nine yeast strains were isolated from the surfaces of plant leaves collected across different regions of China during a yeast diversity survey conducted between 2023 and 2024. Four novel *Phyllozyma* species—*P. aucubae* sp. nov., *P. camelliae* sp. nov., *P. diaoluoensis* sp. nov., and *P. guizhouensis* sp. nov.—were discovered among these strains based on multi-locus (ITS, LSU, *TEF1*, and *RPB1*) and single-locus (ITS) analyses. Our phylogenetic results are consistent with previous observations (Li et al. 2020) and provide further insight into the taxonomy of *Phyllozyma*.

The genus *Phyllozyma* is known as a representative group of ballistosporous yeasts. Most species of the genus generally produce ballistoconidia, which are detected as an opaque mirror image of the culture formed by discharged spores on the lid of an inverted Petri dish (do Carmo-Sousa and Phaff 1962; Kurtzman et al. 2011). However, the production of ballistoconidia is influenced by cultivation methods and varies from clone to clone (Nakase et al. 1993; Nakase 2000). In this study, the two strains of the new species *P. aucubae* sp. nov. were identified as nonballistoconidium-forming yeasts. This phenomenon is extremely rare in the genus *Phyllozyma*, with only *P. jiayinensis* reported to lack the ability to produce ballistoconidia (Li et al. 2020).

Species of *Phyllozyma* are primarily associated with plant substrates and have thus far been isolated only in their yeast morphs, particularly from phyllospheric environments (Nakase and Suzuki 1985; Nakase et al. 1994; Furuya et al. 2012; Li et al. 2020). In this study, the nine strains, characterized as belonging to four new *Phyllozyma* species, were also associated with plant leaves, like most other species in the genus. *P. aucubae* sp. nov. was repeatedly recovered from the same plant, *Aucuba japonica*, collected at East Mountain Park. *P. diaoluoensis* sp. nov. was isolated from three different plants gathered on Diaoluo Mountain. *P. camelliae* sp. nov. and *P. guizhouensis* sp. nov. were found on different plants collected from two different mountains. These findings further support the hypothesis that *Phyllozyma* species are widely distributed in association with plant surfaces. Although the ecology of *Phyllozyma* remains largely uncharacterized, it has been speculated—based on habitat associations and morphological traits—that their propagules may originate from meiosporangia of unknown mycoparasitic basidiomycetes inhabiting leaves or neighboring twigs. The presence of ballistoconidia may facilitate aerial dispersal between fungal hosts and plant surfaces (Oberwinkler 2017). Furthermore, recent work by Schoutteten et al. (2023) demonstrated that *Slooffia*, previously known exclusively in its yeast state, possesses a mycoparasitic filamentous morph. This raises the possibility that *Phyllozyma* species may also be dimorphic, with an as-yet-undiscovered hyphal stage. Given that filamentous morphs in Basidiomycota are typically associated with basidium formation and sexual reproduction, mating assays involving compatible yeast strains may provide insights into their sexual cycle (Schoutteten et al. 2024). Additional efforts, including pure culture isolation and co-cultivation experiments, are warranted to uncover the putative filamentous stage and to elucidate the full life cycle of *Phyllozyma*.

As a result of this study, 13 species are currently assigned to the genus *Phyllozyma*. Among them, *P. producta* and *P. corallina* have been reported as pathogens causing pseudo-greasy spot of citrus (Furuya et al. 2012). Consequently, interest in these yeasts extends beyond their taxonomy to include their ecosystem function, pathogenicity, and potential agricultural, industrial, and medical applications of economic value.

## Supplementary Material

XML Treatment for Phyllozyma aucubae

XML Treatment for Phyllozyma camelliae

XML Treatment for Phyllozyma diaoluoensis

XML Treatment for Phyllozyma guizhouensis

## ﻿Additional information

### Use of AI

No use of AI was reported.

### Author ORCIDs

Zhi-Wen Xi https://orcid.org/0000-0002-5814-5283

Chun-Yue Chai https://orcid.org/0000-0003-0284-5560

Qiu-Hong Niu https://orcid.org/0000-0003-1695-7117

Feng-Li Hui https://orcid.org/0000-0001-7928-3055

### Data availability

All of the data that support the findings of this study are available in the main text or Supplementary Information.

## Supplementary materials

Supplementary material 1 A dataset of ITS, LSU, *TEF1*, and *RBP1* for Fig. 1 Data type fas https://binary.pensoft.net/file/1440591This dataset is made available under the Open Database License (http://opendatacommons.org/licenses/odbl/1.0/). The Open Database License (ODbL) is a license agreement intended to allow users to freely share, modify, and use this Dataset while maintaining this same freedom for others, provided that the original source and author(s) are credited.Zhi-Wen Xi, Chun-Yue Chai, Qiu-Hong Niu, Feng-Li Hui

Supplementary material 2 A dataset of ITS for Fig. 2 Data type fas https://binary.pensoft.net/file/1440592This dataset is made available under the Open Database License (http://opendatacommons.org/licenses/odbl/1.0/). The Open Database License (ODbL) is a license agreement intended to allow users to freely share, modify, and use this Dataset while maintaining this same freedom for others, provided that the original source and author(s) are credited.Zhi-Wen Xi, Chun-Yue Chai, Qiu-Hong Niu, Feng-Li Hui
